# Monkeypox after Occupational Needlestick Injury from Pustule

**DOI:** 10.3201/eid2812.221374

**Published:** 2022-12

**Authors:** João P. Caldas, Sofia R. Valdoleiros, Sandra Rebelo, Margarida Tavares

**Affiliations:** Centro Hospitalar Universitário de São João, Porto, Portugal (J.P. Caldas, S.R. Valdoleiros, S. Rebelo, M. Tavares);; University of Porto, Porto (J.P. Caldas, S.R. Valdoleiros, S. Rebelo, M. Tavares);; European Society for Clinical Microbiology and Infectious Diseases, Basel, Switzerland (S.R. Valdoleiros);; Directorate-General of Health, Lisbon, Portugal (M. Tavares)

**Keywords:** monkeypox, monkeypox virus, MPXV, viruses, needlestick injury, emerging communicable diseases, occupational exposure, pustule, injuries, postexposure prophylaxis, zoonoses, Portugal

## Abstract

We report a case of monkeypox in a physician after an occupational needlestick injury from a pustule. This case highlights risk for occupational transmission and manifestations of the disease after percutaneous transmission: a short incubation period, followed by a solitary lesion at the injured site and later by systemic symptoms.

The 2022 multicountry monkeypox outbreak has been linked primarily to intimate sexual contact. Although there are reported cases among healthcare workers (HCW), most are described to have been acquired in the community setting (*1*). We report a case of monkeypox after an occupational needlestick injury.

## The Study

In late July 2022, a healthy 29-year-old male physician from the Infectious Diseases Department of a tertiary hospital in Portugal had a needlestick injury in the left index finger with a needle used to collect a fluid sample from a man who had a pustular rash, later confirmed to be monkeypox. The physician punctured a pustule with the needle because he was unable to obtain material by swabbing it. He was wearing the recommended personal protective equipment; the gloves appeared intact to him, and there was no wound or bleeding. Thus, he did not report the incident as an occupational exposure accident or considered it for postexposure vaccination. No other risk factors for monkeypox were present.

**Figure 1:**
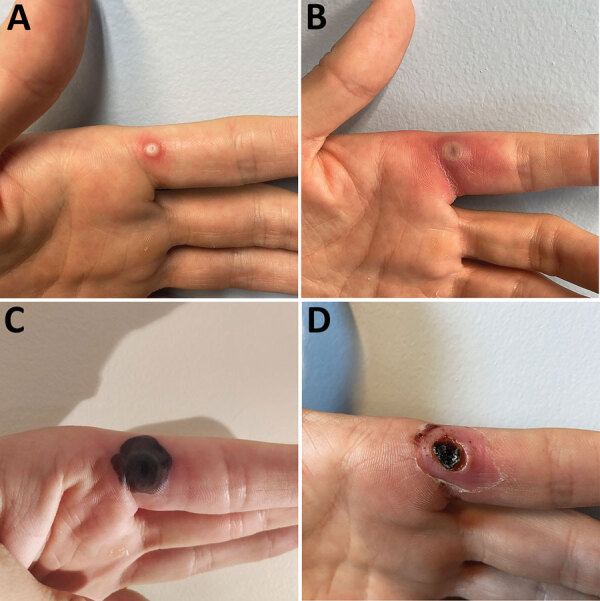
Progress of monkeypox lesion on the finger of a previously healthy male physician in Portugal after occupational needlestick injury from pustule. A) Index lesion on the fourth day of illness. B) Index lesion on the sixth day of illness. C) Index lesion on the 18th day of illness. D) Necrotic scab underneath the devitalized tissue of the index lesion on the 24th day of illness.

Four days later, a vesicle appeared on the pricked finger (Figure 1, panel A), and monkeypox virus (MPXV) was identified in its fluid by PCR, showing a cycle threshold (Ct) of 28. The HCW was sent on sick leave, with indication for the institution of contact and droplet isolation measures at home.

No other lesions or symptoms developed during the next 5 days. PCR results for MPXV in the samples collected from the oropharynx and blood were negative on the seventh day after exposure. The case-patient was considered not eligible for postexposure prophylaxis with the modified vaccinia virus Ankara Bavarian Nordic vaccine (https://www.bavarian-nordic.com) because the HCW already had a lesion that contained MPXV.

**Figure 2:**
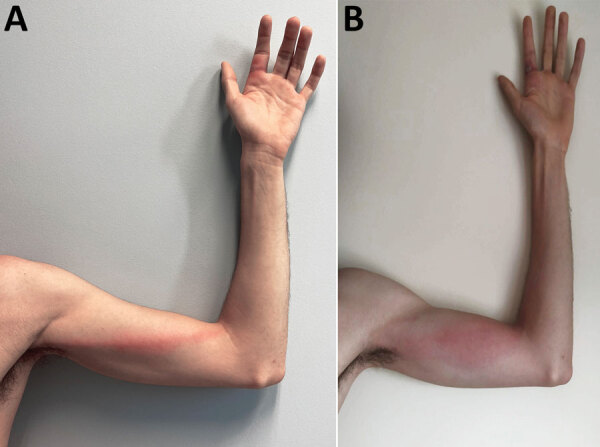
Monkeypox signs in a previously healthy male physician in Portugal after occupational needlestick injury from pustule. A) Tender, indurated, erythematous, and well-delimited linear streak from the left finger to the armpit, on the seventh day of illness. B) Aggravated lymphangitis on the ninth day of illness.

On the sixth day of illness, fever (temperature 38.4°C), chills, and malaise developed and lasted for ≈48 hours. The finger lesion became pustular and painful and showed surrounding erythema and swelling (Figure 1, panel B). A tender, indurated, erythematous and well-delimited linear streak from the left finger to the armpit appeared on the seventh day (Figure 2, panel A), without regional adenopathy. MPXV PCR was repeated in samples from the oropharynx and blood; again, results were negative.

On the eighth day and for the next 3 days, vesicles developed on the scalp, neck, forearm, first finger from both hands and fifth left finger, scrotum, and ankle. A painless right cervical adenopathy also appeared. Despite the absence of leukocytosis or elevation of C-reactive protein level, a bacterial superinfection was assumed because of worsening of the inflammatory signs of the left index finger and of the arm lymphangitis (Figure 2, panel B), and a course of 5 days of oral flucloxacillin was administered. The patient showed clinical improvement. Treatment with tecovirimat was discussed but considered unnecessary given the benign evolution (no mucosal involvement, <10 lesions) and the absence of concurrent conditions.

By the 18th day of illness, all skin lesions, except the one on the left index finger, had evolved through the pustular stage into crust. The index lesion became necrotic (Figure 1, panel C) and was debrided on the 24th day of illness, showing a necrotic scab that had a diameter of 0.5 cm underneath the devitalized tissue (Figure 1, panel D). An MPXV PCR result was still positive in the crust and showed a Ct of 23, but viral culture was negative.

## Conclusions

Several aspects of this case should be emphasized. First, it clearly exemplifies the risks of using sharp instruments for monkeypox testing, which is not recommended. Samples should be collected by vigorously swabbing the surface of lesions or by removing crusts with a forceps or other blunt-tipped sterile instruments (*2*). Unroofing, aspiration of lesions, or otherwise use of sharp instruments before swabbing is not necessary or recommended because of risk for injury from sharp instruments (*2*). The presence of material on the swab surface is indicative of an adequate collection, although it might not always be visible (*2*). For this case-patient, the physician was not certain of an adequate collection after swabbing the lesion, which led him to use a needle for sample collection. If sharp instruments are deemed fully essential for testing, their use, as an exception, should be performed with extreme care, and sharps should be discarded into an adequate container.

Second, this case should remind HCWs about the need to report needlestick injuries and other exposures promptly, regardless of a self-notion of absence of risk, to avoid missing opportunities for postexposure prophylaxis. Penetrating injury is considered a high-risk exposure and an indication for postexposure prophylaxis (*3**,**4*).

Third, the case alerts us to a possible different manifestation and evolution of monkeypox. This difference is especially true when the transmission route is percutaneous. In this case, a solitary lesion developed at the injured site after a short incubation period, followed a few days later by systemic symptoms and the characteristic rash.

Fourth, because of this different evolution, more evidence is needed for decision-making on the timing of postexposure vaccination and use of tecovirimat in similar cases. Despite its benign course, monkeypox might lead to prolonged work absenteeism because international guidelines recommend precautions remain in place until lesions have crusted, scabs have fallen off, and a fresh layer of skin has formed underneath (*5*), which might take several weeks. Thus, it might be reasonable to consider these therapeutic options to prevent or modify the disease course. In this case, when postexposure prophylaxis was discussed, the HCW was not considered eligible for postexposure vaccination because a lesion containing MPXV was already present, which is in accordance with the UK Health Security Agency and the Centers for Disease Control and Prevention recommendations (*3*,*6*). However, more evidence is needed in this field.

Fifth, it is useful to study the infectiousness of the scabs because their dropping off is considered a determinant for deisolation criteria. Pittman et al. verified that monkeypox scabs contained large quantities of viral DNA until and including when they fell off, but viral infectivity of specimens was not determined (*5*). In the case we describe, despite the positive PCR result, with an even lower Ct than the original lesion, the viral culture was negative. Therefore, persistence of positive PCR results might not be a reliable indicator of contagiousness and might lead to prolonged and unnecessary isolation.

Sixth, this case increases awareness of the need for accelerating production of vaccines and increasing their preexposure and postexposure availability. This activity is especially useful for HCWs who deal with monkeypox cases to provide adequate protection and security.

In summary, we report a case of monkeypox in an HCW after a needlestick injury. HCWs should be aware of the risk for transmission of MPXV while infected patients are being evaluated and tested, should report needlestick or other injuries promptly, and should take advantage of available preexposure and postexposure prophylaxis.
